# Preventable causes of cancer in Texas by race/ethnicity: tobacco smoking

**DOI:** 10.4178/epih.e2021046

**Published:** 2021-07-13

**Authors:** Franciska J. Gudenkauf, Aaron P. Thrift

**Affiliations:** 1 Section of Epidemiology and Population Sciences, Department of Medicine, Baylor College of Medicine, Houston, TX, USA; 2 University of Texas Health Science Center at Houston School of Public Health, Houston, TX, USA; 3 Dan L Duncan Comprehensive Cancer Center, Baylor College of Medicine, Houston, TX, USA

**Keywords:** Population attributable fraction, Health status disparities, Risk factors, Tobacco use, Cancer

## Abstract

**OBJECTIVES:**

Tobacco smoking is classified as carcinogenic to humans (International Agency for Research on Cancer Group 1). We aimed to estimate the percentage and number of incident cancer cases diagnosed in Texas in 2015 that were attributable to tobacco smoking, and we examined differences in the proportions of smoking-attributable cancers between the major racial/ethnic subgroups of the population.

**METHODS:**

We calculated population-attributable fractions for cancers attributable to tobacco smoking using prevalence data from the Texas Behavioral Risk Factor Surveillance System and relative risks associated with smoking status from pooled analyses of cohort studies or meta-analyses. Cancer incidence data were collected from the Texas Cancer Registry.

**RESULTS:**

We estimated that 19,000 excess cancer cases or 18.4% of all cancers diagnosed in 2015 in Texans aged ≥ 25 years were caused by tobacco smoking. Males had a higher overall proportion of cancers attributable to tobacco smoking than females (male, 23.3%, 11,993 excess cases; female, 13.5%, 7,006 cases). Approximately 20% of cancer cases in non-Hispanic Whites and non-Hispanic Blacks were attributable to tobacco smoking compared to 12.8% among Hispanics.

**CONCLUSIONS:**

Despite ongoing public health campaigns combatting tobacco use, this preventable behavior still contributes significantly to cancer incidence in Texas. Racial/ethnic differences in smoking prevalence and smoking-attributable cancer incidence should be considered when designing cancer prevention programs.

## INTRODUCTION

As part of its mission, the International Agency for Research on Cancer (IARC) strives to identify causes of cancer [1]. In its *Monographs on the Evaluation of Carcinogenic Risks to Humans*, the IARC classified tobacco smoking as a Group 1 carcinogen, indicating “sufficient evidence of carcinogenicity in humans [1].” Specifically, the IARC declared that tobacco smoking causes cancers of the lung, oral cavity, pharynx, nasal cavity/accessory sinuses, larynx, esophagus, stomach, pancreas, colorectum, liver, kidney (body and pelvis), ureter, urinary bladder, cervix, ovary (mucinous), and myeloid leukemia [1]. Despite aggressive public health attention and declining rates of tobacco use in the United States, smoking remains a concerning source of the burden of cancer and other chronic diseases [2]. Additionally, the smoking prevalence in the United States has historically differed among racial/ethnic groups, suggesting a need to better understand how smoking-attributable cancers are distributed by race/ethnicity [2]. This may better inform cancer prevention while addressing cancer health inequalities.

In the current analysis, we aimed to estimate the population-attributable fractions (PAFs) and number of cancer cases diagnosed in Texas in 2015 that were attributable to tobacco smoking. Furthermore, we hypothesized that differences in PAFs would be apparent across racial/ethnic subgroups given the population diversity in Texas, so we stratified our study by race/ethnicity to expose any possible effect modification that may better guide cancer prevention efforts.

## MATERIALS AND METHODS

The Texas Cancer Registry provided counts of invasive cancer cases newly diagnosed in Texas in 2015, overall and by age group (25-34, 35-44, 45-54, 55-64, 65-74, 75-84, ≥ 85 years), sex, and race/ethnicity (all races/ethnicities, non-Hispanic Whites, non-Hispanic Blacks, Hispanics, and other races/ethnicities) [3]. Coding for cancer sites was performed using the Surveillance, Epidemiology, and End Results Site Recode International Classification of Diseases (ICD)-O-3/World Health Organization 2008 definitions [4]. This code for the lung cancer site also included bronchial cancers [4]. Ovarian mucinous cancer was specified by histology codes [5,6], and nasal cavity/accessory sinus cancers were coded using primary ICD-O-3 site codes [4,7]. Due to available risk estimates, we grouped oral cavity and pharyngeal cancers together, as well as kidney (body and pelvis) and ureteral cancers, and nasal cavity and accessory sinus cancers (Table 1).

Relative risk (RR) estimates for smoking status (current or former, relative to never smokers) were primarily derived from a pooled analysis of United States-based cohort studies examining the association of smoking with the risk of cancer mortality [8]. We elected to use these RR estimates, rather than RR estimates associated with cancer incidence, so that our findings would be both directly comparable and add to (by providing race/ethnicspecific PAFs) findings from a recent study of the overall PAF in the United States [9]. For the majority of cancer sites, there is overlap of the 95% confidence intervals in RR estimates of cancer incidence and mortality associated with smoking status. For cervical cancer and nasal cavity/accessory sinus cancer, estimates from this source were excluded; instead, estimates were obtained from a large prospective cohort study [10] and global meta-analysis [11], respectively, examining the association between smoking and risk of cancer. Table 1 shows the RR estimates used in the current study.

The Texas Behavioral Risk Factor Surveillance System (BRFSS) provided weighted prevalence estimates (weighted to demographic proportions within the population via post-stratification) [12] of current and former smoking in Texas by age group, sex, and race/ethnicity (Table 2) [13]. Current smokers were defined as having smoked 100 cigarettes in their lifetime and currently smoking some days or every day, while former smokers were defined as having smoked 100 cigarettes in their lifetime and not currently smoking [14]. The estimated latency period for cancers attributable to smoking varies widely across studies and across cancer sites [15]; however, because ranges for the latency period include 10 years for nearly all cancer sites, and for consistency across cancer sites, we estimated an average latency period of 10 years for all cancer types [15]. Therefore, we used BRFSS prevalence data from the year 2006.

The following formula was used to calculate PAFs for each cancer type by sex, age group, and race/ethnicity: $PAF=\frac{\Sigma (p_{x}*ERR_{x})}{1+\Sigma (p_{x}*ERR_{x})}$, where *p_x_* is the proportion of the population in exposure category *x* (current or former smoker) and *ERR_x_* is the excess relative risk (RR_x_-1) [16]. We multiplied PAFs by incident cancer counts to determine the number of cancer cases attributable to smoking, stratified by age group, sex, and race/ethnicity. To accommodate the latency period of 10 years, we paired the prevalence data and corresponding PAFs in each age group with cancer incidence data in the age group 10 years older (e.g., 2006 prevalence data for the age group of 25-34 years corresponded to 2015 cancer counts for the age group of 35-44 years). We calculated the age-weighted percentages of incident cancer cases attributable to tobacco smoking by cancer type, sex, and race/ethnicity. Finally, we added all attributable cases across all cancer sites to estimate the percentage of all incident cancers (excluding basal cell carcinoma [BCC] and squamous cell carcinoma [SCC] of the skin) diagnosed in 2015 in Texans aged ≥ 25 years that were attributable to tobacco smoking.

### Ethics statement

This study was conducted according to the guidelines laid down in the Declaration of Helsinki. All data were publically available. The research activities of this study were deemed as exempt by Baylor College of Medicine Ethics Committee as data were deidentified and aggregated. A waiver of consent was obtained because the study was a retrospective analysis and all data were aggregated and de-identified.

## RESULTS

Nearly half of Texan males (47.9%) and a third of Texan females (31.7%) aged ≥ 18 years in 2006 reported having ever smoked tobacco. Overall, 39.8% of Texans in 2006 were self-reported current or former smokers. The prevalence of smoking was highest in persons aged 55-64 years (55.3%) and lowest in persons aged 25-34 years (26.6%) (Table 2). In all age groups, males had a higher prevalence of current or former smoking than females.

Non-Hispanic Whites comprised the racial/ethnic subgroup with the highest prevalence of current or former smoking at 44.3% (Table 2). Meanwhile, the prevalence of tobacco smoking was 37.9% in non-Hispanic Blacks and 30.3% in Hispanics. This trend persisted when stratified by sex; non-Hispanic White male (49.8%) and non-Hispanic White female (38.3%) had higher prevalence rates of tobacco smoking than other racial/ethnic-sex subgroups.

Excluding BCC and SCC of the skin, there were 103,408 cancer cases diagnosed in Texan adults aged ≥ 25 years in 2015, with 51,472 cases in males and 51,936 cases in females. Non-Hispanic Whites had the most cancers (65,214 cases), followed by Hispanics (22,642 cases) and non-Hispanic Blacks (12,020); the remaining cases were found in the other races/ethnicities category (3,532 cases). Overall, 19,000 cancer cases or 18.4% of all incident cancers (excluding BCC and SCC of the skin) diagnosed in 2015 were attributable to tobacco smoking (Table 3). Males had a higher overall proportion of cancers attributable to tobacco smoking than females (male, 23.3%, 11,993 excess cases; female, 13.5%, 7,006 cases). The cancer site with the highest PAF was the lung/bronchus (84.7%), followed by the larynx (77.9%) and esophagus (52.6%). Males had higher site-specific PAFs than females for all cancer sites except the larynx and pancreas (excluding female-specific cancers, mucinous ovarian cancer and cervical cancer).

Stratification by racial/ethnic subgroups revealed that non-Hispanic Whites (20.2%, 13,152 excess cases) and non-Hispanic Blacks (20.1%, 2,416 excess cases) had higher proportions of all cancers attributable to tobacco smoking than Hispanics (12.8%, 2,902 excess cases). Males had higher overall PAFs than females across all racial/ethnic subgroups. For non-Hispanic Whites and Hispanics, the lung/bronchus, larynx, and esophagus were the cancer sites that contributed the highest proportions of excess cases, consistent with the findings for all races/ethnicities combined. However, for non-Hispanic Blacks, the lung/bronchus, larynx, and oral cavity/pharynx had the highest site-specific PAFs. While we found that males had higher site-specific PAFs than females for all cancer sites except the larynx and pancreas (and excluding female-specific cancers) for all races/ethnicities combined, this was not the case within racial/ethnic groups. In non-Hispanic Whites, females had higher PAFs for the larynx and pancreas sites, while the PAFs were equal between the sexes for the oral cavity/pharynx. In non-Hispanic Blacks, females had higher site-specific PAFs for the lung/bronchus, larynx, esophagus, pancreas, colorectum, and urinary bladder, and there was no sex difference for nasal cavity/accessory sinuses. In Hispanics, the larynx was the only site with a higher PAF in females.

## DISCUSSION

In this study, we estimated that in 2015 in Texans aged ≥ 25 years, 19,000 excess cancer cases or 18.4% of all incident cancer cases (excluding BCC and SCC of the skin) were attributable to tobacco smoking. As expected, the cancer site that contributed the highest proportions of cancers was the lung/bronchus (84.7%); tobacco smoking also caused a high fraction of laryngeal (77.9%) and esophageal (52.6%) cancer cases in Texas. Males (23.3%) had a higher proportion of cancers attributable to tobacco smoking than females (13.5%). Non-Hispanic Whites (20.2%) and non-Hispanic Blacks (20.1%) had higher percentages of smoking-attributable cancers than Hispanics (12.8%).

Our findings are comparable to those of a similar PAF study conducted recently in the United States overall [9], which estimated that 19.0% of cancers (excluding non-melanoma skin cancers) were attributable to tobacco smoking in the United States in 2014 in persons aged ≥ 30 years. However, our results and the United States-wide results are notably higher than studies conducted in other countries; in Australia in 2010 [16], 13.3% of all cancers were found to be attributable to tobacco smoking, while in the United Kingdom in 2015 [17], 15.1% of all cancers were due to tobacco smoking. This may be a reflection of differences in study methodology, estimates of smoking prevalence, and sources used for RR estimates. Nonetheless, these data suggest that at least 1 in 8 cancer cases are attributable to tobacco smoking in Western populations. By sex, we found a higher percentage of smoking-attributable cancers in males (23.3%) than in females (13.5%), a pattern consistent with the studies conducted in the United States overall (male, 23.6%; female, 14.5%) [9], Australia (male, 15.8%; female, 10.0%) [16], and the United Kingdom (male, 17.7%; female, 12.4%) [17].

Our analysis exposes differences in PAFs across major racial/ethnic subgroups of the population, with greater proportions of smoking-attributable cancers in non-Hispanic Whites (20.2%) and non-Hispanic Blacks (20.1%) than in Hispanics (12.8%). The higher prevalence rates of current or former smoking in non-Hispanic Whites (44.3%) and non-Hispanic Blacks (37.9%) than in Hispanics (30.3%) likely contributed to the higher PAFs in non-Hispanic Whites and non-Hispanic Blacks. However, it should be noted that while the overall PAFs for non-Hispanic Whites and non-Hispanic Blacks were nearly equal, the prevalence of smoking in non-Hispanic Whites was about 1.17-fold higher than in non-Hispanic Blacks. This may suggest that a greater proportion of cancers caused by factors other than smoking contributed to the denominator of overall cancer incidence in non-Hispanic Whites, thus diluting the overall PAF to an estimate that is lower than expected based solely on the contribution of smoking prevalence.

The analysis of site-specific PAFs revealed that the lung/bronchus (84.7%), larynx (77.9%), and esophagus (52.6%) sites contributed the highest fractions of smoking-attributable cancers in Texas (all sexes, all races/ethnicities). Studies from the United States overall in 2014 (lung, 81.7%; larynx, 73.8%; esophagus, 50.0%) [9], Australia in 2010 (lung, 79.6%; larynx, 77%; esophagus, 60%) [16], and the United Kingdom in 2010 (results not reported for all persons, only reported by sex) [18] also reported these same 3 sites as the top contributors to cancer incidence, with similar magnitudes of PAFs. However, it should be noted that some of these studies used different methodologies to derive PAFs for lung cancer, so PAFs may not be directly comparable [16,18]. These findings further emphasize that tobacco smoking serves as an important risk factor, especially for cancers of the lung, larynx, and esophagus.

There are several limitations to our study. First, we primarily derived RR estimates from a pooled analysis of cohort studies, which provided RRs for cancer mortality for persons aged ≥ 55 years instead of cancer incidence [8]. While it can be argued that estimates based on cancer incidence instead of mortality would have been more appropriate, we elected to reference the same source as a similar study [9] to ensure comparability of findings, which we consider a strength of this study. Additionally, we used prevalence estimates for self-reported current and former smoking, which were defined as having smoked 100 cigarettes in one’s lifetime and currently smoking some/every day(s) versus not, respectively [14]. However, it is known that smoking has a cumulative effect on cancer risk based on the amount and period of time smoked [16], which is traditionally denoted by pack-years. Thus, even within each of our categories for current smokers and former smokers, individuals would likely have differences in risk due to differences in pack-years smoked. Therefore, perhaps a more robust study could use prevalence data based on pack-years and RR estimates based on dose-response by pack-years, although—to the best of our knowledge—this type of surveillance and comprehensive risk assessment is currently unavailable. Further, this complexity has stimulated the use of different methods to calculate PAFs for lung cancer in other studies, which presumably bypass these shortcomings [16,18]. However, we maintained standard methods and formulae for all cancer sites when calculating PAFs. Also, we assumed a latency period of 10 years between exposure and outcome, although the latency periods are difficult to measure and likely differ by cancer site [15]. Lastly, we caution against using the other races/ethnicities category to draw inferences about racial/ethnic groups not specifically analyzed in this study, as this group was small when stratified and may not be representative of other racial/ethnic identities.

In summary, we estimated that 18.4% of all cancers (19,000 excess cancer cases) diagnosed in Texas in 2015 were attributable to tobacco smoking. Further, non-Hispanic Whites (20.2%) and non-Hispanic Blacks (20.1%) had higher percentages of smoking-attributable cancers than Hispanics (12.8%). In response to the high burden of cancers caused by tobacco smoking, public health campaigns combatting tobacco use should continue to stress the causal relationship between smoking and cancer, while also directing attention toward racial/ethnic differences, which may help in tailoring cancer prevention efforts more appropriately.

## Figures and Tables

**Table 1.** Relative risks (RRs) associated with smoking by status (current vs. never-smokers, former vs. never-smokers)

Cancer site	Exposure category (smoker)	Males	Females
Lung, bronchus^1^	Current	25.3	22.9
	Former	6.8	6.8
Oral cavity, pharynx^2^	Current	5.7	5.6
	Former	1.7	2.2
Larynx	Current	13.9	103.8
	Former	2.4	11.6
Esophagus	Current	3.9	5.1
	Former	2.6	2.2
Stomach	Current	1.9	1.7
	Former	1.5	1.1^7^
Pancreas	Current	1.6	1.9
	Former	1.0	1.2
Colorectum	Current	1.4	1.6
	Former	1.2	1.2
Liver	Current	2.3	1.8
	Former	1.5	1.1^7^
Kidney, renal pelvis, ureter^3^	Current	1.8	1.2^7^
	Former	1.5	1.2^7^
Urinary bladder	Current	3.9	3.9
	Former	2.4	2.3
Cervix	Current	-	1.9
	Former	-	1.5
Ovary (mucinous; histology codes: 8470,8471,8480,8481)^4^	Current	-	1.1^7^
	Former	-	1.1^7^
Myeloid leukemia^5^	Current	1.9	1.1^7^
	Former	1.4	1.1^7^
Nasal cavity, accessory sinuses (primary site codes: C300,C310-319)^6^	Current	1.95	1.95
	Former	1.39	1.39

1 RR for trachea, lung, bronchus.

2 RR for lip and oral cavity.

3 RR for kidney and renal pelvis.

4 RR for ovary.

5 RR for acute myeloid leukemia.

6 RR for nasal-sinuses, nasopharynx.

7 The reported confidence interval includes the null value.

**Table 2.** Prevalence (%) of tobacco smoking^1^ in Texans aged ≥18 years in 2006, overall and by race/ethnicity and age group

Variables		Males			Females			Persons		
		Current smoker	Former smoker	Both	Current smoker	Former smoker	Both	Current smoker	Former smoker	Both
All		20.6	27.3	47.9	15.6	16.1	31.7	18.1	21.7	39.8
Race/ethnicity										
	Non-Hispanic Whites	17.3	32.5	49.8	17.8	20.5	38.3	17.6	26.7	44.3
	Non-Hispanic Blacks	34.3	15.2	49.5	20.0	10.4	30.4	25.6	12.3	37.9
	Hispanics	24.9	18.9	43.8	8.9	9.5	18.4	16.4	13.9	30.3
	Other races/ethnicities	20.8	21.0	41.8	22.8	11.5	34.3	21.7	17.0	38.7
Age (yr)										
	18-24	31.3	10.7	42.0	14.6	7.2	21.8	23.4	9.0	32.4
	25-34	18.4	12.2	30.6	12.8	9.8	22.6	15.6	11.0	26.6
	35-44	18.5	21.4	39.9	19.8	12.9	32.7	19.1	17.2	36.3
	45-54	24.1	32.7	56.8	20.4	16.1	36.5	22.3	24.5	46.8
	55-64	18.3	50.6	68.9	16.3	26.3	42.6	17.3	38.0	55.3
	65-74	11.4	51.1	62.5	10.5	26.0	36.5	10.9	37.5	48.4
	75-84	7.5	57.5	65.0	7.0	29.7	36.7	7.2	39.8	47.0
	≥85	21.2	51.7	72.9	6.8	24.7	31.5	13.2	36.7	49.9

1 Current smoker: every day or some days+smoked 100 cigarettes in lifetime; Former smoker: smoked 100 cigarettes in lifetime+not currently smoking.

**Table 3.** Age-weighted population-attributable fractions (PAFs) of cancers attributable to smoking in Texas in 2015 by race/ethnicity (%), adults aged ≥25 years

Variables		Lung, bronchus	Oral cavity, pharynx	Larynx	Esophagus	Stomach	Pancreas	Colorectum	Liver	Kidney, renal pelvis, ureter	Urinary bladder	Cervix	Ovary (mucinous)	Myeloid leukemia	Nasal cavity, accessory sinuses	All cancers PAF (excess cases)^1^
All																
	Males	86.9	53.4	74.3	54.2	26.5	9.3	13.1	30.8	25.3	52.5	-	-	23.9	24.3	23.3 (11,993)
	Females	81.9	49.2	94.9	46.5	11.3	15.0	11.9	12.8	6.8	41.3	17.8	3.1	3.4	18.7	13.5 (7,006)
	Persons	84.7	52.3	77.9	52.6	20.2	12.1	12.6	26.1	18.2	49.8	17.8	3.1	15.1	22.5	18.4 (19,000)
Non-Hispanic Whites																
	Males	86.5	51.2	72.5	53.8	26.6	8.1	12.9	29.6	25.2	52.4	-	-	23.8	24.3	24.4 (8,076)
	Females	82.6	51.2	95.2	48.1	11.7	15.7	12.6	13.4	7.5	42.9	20.8	3.7	3.9	19.8	15.8 (5,076)
	Persons	84.7	51.2	77.3	52.7	21.0	11.7	12.8	25.4	18.8	50.2	20.8	3.7	15.4	22.6	20.2 (13,152)
Non-Hispanic Blacks																
	Males	87.5	62.1	76.8	53.2	28.1	16.9	15.0	39.0	27.3	51.0	-	-	27.3	28.0	24.4 (1,463)
	Females	87.6	56.7	96.4	58.1	17.0	22.9	16.3	19.3	8.4	51.8	20.2	4.2	4.1	28.0	15.8 (953)
	Persons	87.5	60.3	80.2	54.8	23.1	20.1	15.6	34.2	19.6	51.3	20.2	4.2	16.0	28.0	20.1 (2,416)
Hispanics																
	Males	85.8	53.8	73.1	53.3	25.8	10.0	13.1	31.3	24.7	51.0	-	-	23.6	24.3	19.6 (2,092)
	Females	72.0	36.7	84.9	34.7	7.7	9.4	8.2	8.3	4.6	27.0	11.6	1.9	8.5	14.9	6.8 (810)
	Persons	80.1	49.0	73.9	50.3	17.7	9.8	11.1	25.0	16.4	45.1	11.6	1.9	17.5	22.3	12.8 (2,902)
Other races/ethnicities																
	Males	83.2	50.6	63.7	47.3	22.3	9.3	11.9	27.9	22.0	43.9	-	-	21.3	20.7	17.7 (301)
	Females	78.1	49.3	18.5	38.5	11.6	14.0	12.3	11.5	6.5	34.6	21.0	3.4	3.1	15.8	9.5 (174)
	Persons	81.1	50.1	60.5	46.0	17.9	11.8	12.1	24.0	16.0	42.2	21.0	3.4	11.6	18.9	13.4 (475)

Totals may not sum manually due to rounding.

1 Excluding basal cell carcinoma and squamous cell carcinoma of the skin; All cancers combined are displayed as PAF (excess cases).
